# Clinical verification of body mass index and tumor immune response in patients with breast cancer receiving preoperative chemotherapy

**DOI:** 10.1186/s12885-021-08857-7

**Published:** 2021-10-20

**Authors:** Koji Takada, Shinichiro Kashiwagi, Yuka Asano, Wataru Goto, Sae Ishihara, Tamami Morisaki, Masatsune Shibutani, Hiroaki Tanaka, Kosei Hirakawa, Masaichi Ohira

**Affiliations:** 1 Department of Breast and Endocrine Surgery, Osaka City University Graduate School of Medicine, 1-4-3 Asahi-machi, Abeno-ku, Osaka, 545-8585 Japan; 2 Department of Gastrointestinal Surgery, Osaka City University Graduate School of Medicine, 1-4-3 Asahi-machi, Abeno-ku, Osaka, 545-8585 Japan

**Keywords:** Breast cancer, Body mass index, Tumor-infiltrating lymphocytes, Preoperative chemotherapy, Tumor immune microenvironment

## Abstract

**Purpose:**

The body mass index (BMI) is commonly used as a simple indicator of obesity; patients with early-stage breast cancer who are obese (OB) per BMI measurements have been shown to have high postoperative recurrence and low survival rates. On the other hand, it has been shown that lymphocytes present in the vicinity of malignant growths that are involved in the tumors’ immune responses influence the efficacy chemotherapy. Therefore, we hypothesized that OB patients with breast cancer have a lower density of tumor-infiltrating lymphocytes (TILs), which may influence the therapeutic effect of preoperative chemotherapy (POC). In this study, we measured pretreatment BMI and TILs in patients with breast cancer who underwent POC, examined the correlations between these two factors, and retrospectively analyzed their therapeutic outcomes and prognoses.

**Methods:**

The participants in this study were 421 patients with breast cancer who underwent surgical treatment after POC between February 2007 and January 2019. The patient’s height and weight were measured before POC to calculate the BMI (weight [kg] divided by the square of the height [m^2^]). According to the World Health Organization categorization, patients who weighed under 18.5 kg/m^2^ were classified as underweight (UW), those ≥18.5 kg/m^2^ and > 25 kg/m^2^ were considered normal weight (NW), those ≥25 kg/m^2^ and < 30 kg/m^2^ were overweight (OW), and those ≥30 kg/m^2^ were OB. The TILs were those lymphocytes that infiltrated the tumor stroma according to the definition of the International TILs Working Group 2014.

**Results:**

The median BMI was 21.9 kg/m^2^ (range, 14.3–38.5 kg/m^2^); most patients (244; 64.5%) were NW. Among all 378 patients with breast cancer, the TIL density was significantly lower in OB than in NW and OW patients (vs. NW: *p* = 0.001; vs. OW: *p* = 0.003). Furthermore, when examining patients with each breast cancer type individually, the OS of those with TNBC who had low BMIs was significantly poorer than that of their high-BMI counterparts (log rank *p* = 0.031).

**Conclusions:**

Our data did not support the hypothesis that obesity affects the tumor immune microenvironment; however, we showed that being UW does affect the tumor immune microenvironment.

**Supplementary Information:**

The online version contains supplementary material available at 10.1186/s12885-021-08857-7.

## Background

Obesity has long been cited as a poor prognostic factor in patients with breast cancer [1–4]. The body mass index (BMI) is commonly used as a simple indicator of obesity; patients with early-stage breast cancer who are obese (OB) per BMI measurements have been shown to have high postoperative recurrence and low survival rates. One of the causes for this is that levels of estrogen, insulin, insulin-like growth factor, and cytokines that promote tumor growth are increased in OB patients with breast cancer [5–7]. Another cause is that obesity-associated chronic inflammation and hypoxia are present in tumor tissues [8–10]. Furthermore, there have been some studies in recent years showing that pathological complete response (pCR) rates in OB patients with breast cancer who received chemotherapy remained low owing to the abovementioned factors [11–14].

It has been shown that lymphocytes present in the vicinity of malignant growths that are involved in the tumors’ immune responses influence the efficacy chemotherapy [15–18]. These ‘tumor-infiltrating lymphocytes’ (TILs) have also been reported in patients with breast cancer [19, 20]. Furthermore, it has been reported that the densities of TILs differ depending on the tumor subtype [21]; specifically, their density is higher in hormone receptor-negative breast cancer [22, 23]. While there are few reports of other factors affecting TILs, a role for obesity in tumor immunity has been suggested for some time [24, 25]. However, there are still few published studies of the correlation between BMI and TILs.

Therefore, we hypothesized that BMI affects prognosis because of differences in the immune microenvironment. In this study, we measured pretreatment BMI and TILs in patients with breast cancer who underwent POC, examined the correlations between these two factors, and retrospectively analyzed their therapeutic outcomes and prognoses.

## Methods

### Patients

The participants in this study were 421 patients with breast cancer who underwent surgical treatment after POC at the Osaka City University Hospital between February 2007 and January 2019. All patients were pathologically diagnosed with breast cancer by core needle biopsy or vacuum-assisted biopsy. Afterward, the expression levels of estrogen receptor (ER), progesterone receptor (PgR), human epidermal growth factor receptor 2 (HER2), and Ki67 were evaluated via immunohistochemistry and classified into three subtypes as described previously [26]. Hormone receptor-positive breast cancer (HRBC) was defined as a tumor positive for ER and/or PgR. HER2-enriched breast cancer (HER2BC) was defined as ER-negative, PgR-negative, and HER2-positive. Finally, triple-negative breast cancer (TNBC) was defined as ER-, PgR-, and HER2-negative. Prior to POC, computed tomography, ultrasonography, and bone scintigraphy were used to assess breast cancer progression. The patient’s height and weight were measured before POC to calculate the BMI (weight [kg] divided by the square of the height [m^2^]). According to the World Health Organization categorization, patients who weighed under 18.5 kg/m^2^ were classified as underweight (UW), those ≥18.5 kg/m^2^ and > 25 kg/m^2^ were considered normal weight (NW), those ≥25 kg/m^2^ and < 30 kg/m^2^ were overweight (OW), and those ≥30 kg/m^2^ were OB. The first half of POC consisted of four courses of FEC100 (which includes 500 mg/m^2^ fluorouracil, 100 mg/m^2^ epirubicin, and 500 mg/m^2^ cyclophosphamide) every 3 weeks. In the second half, 12 courses of 80 mg/m^2^ paclitaxel were administered weekly; moreover, weekly (2 mg/kg) or tri-weekly (6 mg/kg) trastuzumab was also administered if the tumor was HER2-positive [27–29]. Imaging was repeated after POC but before surgery to evaluate the therapeutic effect according to the Response Evaluation Criteria in Solid Tumors [30]. Patients with clinical partial and complete responses were defined as “responders” when calculating the objective response rate (ORR), while those assessed to have clinical stable disease and clinical progressive disease were defined as “non-responders”. Either mastectomy or breast-conserving surgery was performed based on the degree of breast cancer progression before and after POC while also considering the patient’s wishes [31]. The definition of a pCR followed the National Surgical Adjuvant Breast and Bowel Project B-18 protocol as “the complete disappearance of the invasive components of the lesion with or without intraductal components, including that in the lymph nodes” [32]. Standard adjuvant therapy was administered to the tumor subtype and chosen surgical procedure. Overall survival (OS) was defined as the interval between surgery and death from any cause, while disease-specific survival (DSS) was defined as the interval between surgery and death from breast cancer. The median follow-up time was 1881 days (range, 63–4551 days) from surgery.

### Histopathological evaluation of TIL density

TIL density was evaluated within the biopsy tissue used to diagnose breast cancer. The TILs were those lymphocytes that infiltrated the tumor stroma according to the definition of the International TILs Working Group 2014 [15]. The density of TILs was calculated from the average of five random fields of view as described by the Working Group [15]. Furthermore, the cutoff value for TIL density was set at 10%, and patients were divided into four groups based on this density (score = 3, > 50%; score = 2, > 10–50%; score = 1, ≤10%; and score = 0, absent TILs) **(Supplementary Fig.** **1****)**, as described previously [33, 34].

### Statistical analysis

All statistical analyses were performed using the JMP version 15 software package (SAS, Tokyo, Japan). The distribution of TIL density according to the BMI category was evaluated using Student’s t-test. Pearson’s chi-square test was used to evaluate the correlation between two groups of clinicopathological features. Analyses of disease-free survival (DFS), OS, and DSS were performed using the Kaplan-Meier method; results were compared using the log-rank test. The hazard ratios (HRs) and 95% confidence intervals were calculated using the Cox proportional hazards model, and multivariable analysis was performed using a Cox regression model. *P*-values < 0.05 were defined as significant.

## Results

### Clinicopathological features

Of the original 421 participants of this study, 43 were excluded because TILs could not be evaluated on their biopsy tissue samples **(**Table 1**)**. The median age of the remaining 378 patients was 56 years (range, 24–78 years). The median tumor diameter was 28.7 mm (range, 9.2–119.8 mm), with skin infiltration present in 60 patients (15.9%). No lymph node metastases were found in 132 of the patients (34.9%). There were 159 patients (42.1%) who had HRBC and 93 (24.6%) who had HER2BC; the remaining 126 patients (33.3%) had TNBC. Three hundred thirty-seven patients responded to treatment; the ORR was 89.2% and the pCR rate was 33.9%. Moreover, 169 patients (44.7%) had a high TIL density. The median BMI was 21.9 kg/m^2^ (range, 14.3–38.5 kg/m^2^); most patients (244; 64.5%) were NW.

**Table 1:** Clinicopathological features of 378 patients who were treated with preoperative chemotherapy

Parameters (*n* = 378)	Number of patients (%)
Age (years old)	56 (24–78)
Tumor size (mm)	28.7 (9.2–119.8)
Skin infiltration Negative / Positive	318 (84.1%) / 60 (15.9%)
Lymph node metastasis N0 / N1 / N2 / N3	132 (34.9%) / 140 (37.0%) / 71 (18.8%) / 35 (9.3%)
Estrogen receptor Negative / Positive	223 (59.0%) / 155 (41.0%)
Progesterone receptor Negative / Positive	301 (79.6%) / 77 (20.4%)
HER2 Negative / Positive	238 (63.0%) / 140 (37.0%)
Ki67 ≤ 14% / > 14%	121 (32.0%) / 257 (68.0%)
Intrinsic subtype HRBC / HER2BC / TNBC	159 (42.1%) / 93 (24.6%) / 126 (33.3%)
Objective response rate Non-Responders / Responders	41 (10.8%) / 337 (89.2%)
Pathological response Non-pCR / pCR	250 (66.1%) / 128 (33.9%)
TILs Low / High	209 (55.3%) / 169 (44.7%)
Body mass index (kg/m2)	21.9 (14.3–38.5)
Body mass index categorized Underweight / Normal / Overweight / Obese	49 (13.0%) / 244 (64.5%) / 66 (17.5%) / 19 (5.0%)

HER: human epidermal growth factor receptor. HRBC: hormone receptor-positive breast cancer (ER+ and/or PgR+). HER2BC: human epidermal growth factor receptor 2-enriched breast cancer (ER-, PgR-, and HER2+). TNBC: triple negative breast cancer (ER-, PgR-, and HER2-). pCR: pathological complete response. TILs: tumor-infiltrating lymphocytes

The correlation between pathological response and clinicopathological factors was examined **(Supplementary Table** **1****)**. In all cases, small tumors (*p* = 0.021) and no skin infiltration (*p* < 0.001) were significantly more likely to achieve pCR. As breast cancer subtypes, ER negative (*p* < 0.001), PgR negative (*p* < 0.001), HER2 positive (*p* = 0.002), and high Ki67 (*p* = 0.005) were significantly more likely to achieve pCR, so HER2BC (p < 0.001) and TNBC (*p* = 0.031) were significantly more likely to have pCR. When examined with HER2BC and TNBC, skin infiltration in HER2BC had a significant effect on pCR (*p* = 0.006).

The correlation between TILs and clinicopathological factors was examined **(Supplementary Table** **2****)**. Compared to patients with breast cancer who had higher TIL densities, those with lower densities more frequently had skin infiltration (*p* = 0.005), ER positivity (*p* < 0.001), PgR positivity (*p* < 0.001), HER2 negativity (*p* = 0.015), and lower Ki67 (*p* < 0.001). Hence, the proportions of patients with HER2BC and TNBC were significantly smaller in the low TIL density group than in the high TIL density group (*p* < 0.001 and *p* = 0.019, respectively). Patients in the low TIL density group had a significantly lower ORR (*p* = 0.001) and pCR rate (*p* < 0.001) than did their counterparts in the high TIL density group. Therapeutic outcomes were significantly poorer among patients with low TILs than among those with high TILs even when patients with TNBC (ORR: *p* = 0.016; pCR rate: *p* = 0.008) and HER2BC (ORR: *p* = 0.023; pCR rate: *p* = 0.018) were analyzed separately.

### Relationship between BMI and TILs

**Fig. 1:**
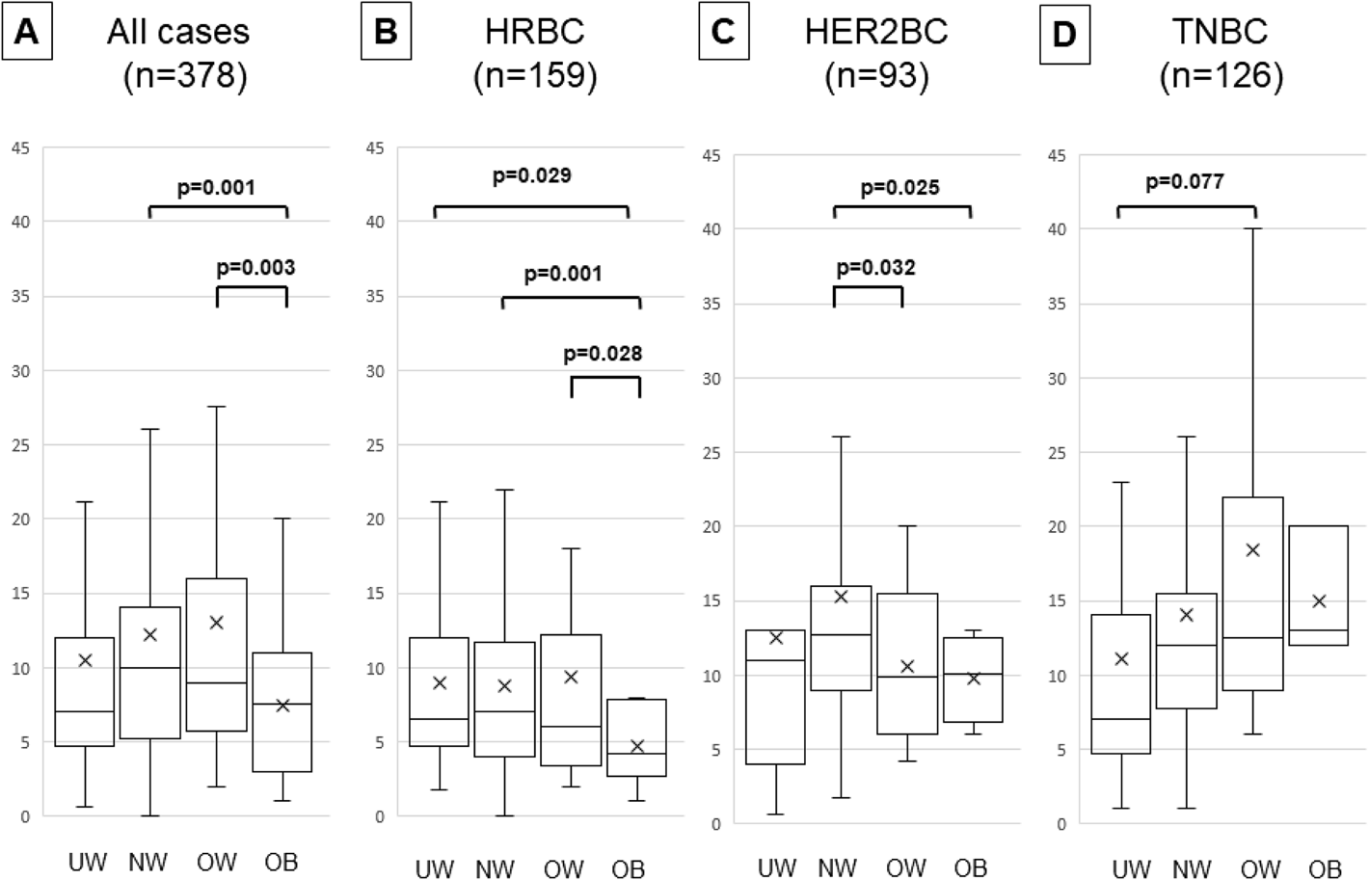
Comparison of tumor-infiltrating lymphocytes (TILs) density by differences in BMI categorized (UW; underweight, NW; normal weight, OW; overweight, OB; obese) by box-plot diagrams. X indicates the average value. All two groups were analyzed by Student’s t-test. Between the two groups not shown is *p* > 0.1. The *p*-value between the two groups without the p-value is greater than 0.1. **A** all case, **B** hormone receptor positive breast cancer (HRBC), **C** HER2-enriched breast cancer (HER2BC), **D** triple-negative breast cancer (TNBC)

Among all 378 patients with breast cancer, the TIL density was significantly lower in OB than in NW and OW patients (vs. NW: *p* = 0.001; vs. OW: *p* = 0.003) **(**Fig. 1**)**. In particular, OB patients with HRBC had significantly lower TIL densities than did those in the other three BMI categories (vs. UW: *p* = 0.029; vs. NW: p = 0.001; vs OW: *p* = 0.028). On the other hand, NW patients with HER2BC had significantly higher TIL densities than did OB and OW patients (vs. OB; *p* = 0.025, vs. OW; *p* = 0.032). However, among patients with TNBC, those who were UW tended to have lower TIL densities than did those who were OW, although the difference was not significant (*p* = 0.077).

### Correlation between BMI and clinicopathological factors

We next examined the correlation between BMI and clinical pathological factors given that our abovementioned data suggested that TIL density may be lower in patients with breast cancer who were UW and OB (Table 2). We found that patients who were UW had a significantly higher frequency of skin infiltration than did those who were NW/OW (*p* = 0.012) while the rate of PgR positivity was not significantly different (*p* = 0.085). Moreover, TIL density tended to be lower in UW patients (*p* = 0.097). Additionally, OB patients had a significantly higher frequency of skin infiltration (*p* = 0.004) and of PgR positivity (*p* = 0.043) than did NW/OW patients. Patients with TNBC tended to be less frequent in the OB group (*p* = 0.098) while TIL density also tended to be lower (*p* = 0.073).

**Table 2:** Difference in clinicopathological features due to body mass index categorized

	Body mass index categorized		*p* value	Body mass index categorized		*p* value
	Underweight (*n* = 49)	Nomal / Overweight (*n* = 310)		Nomal / Overweight(*n* = 310)	Obese(*n* = 19)	
Age (years old)						0.928
≤ 60	33 (67.3%)	209 (67.4%)		209 (67.4%)	13 (68.4%)	
> 60	16 (32.7%)	101 (32.6%)	0.992	101 (32.6%)	6 (31.6%)	
Tumor size (mm)						0.267
≤ 20.0	9 (18.4%)	51 (16.5%)		51 (16.5%)	5 (26.3%)	
> 20.0	40 (81.6%)	259 (83.5%)	0.738	259 (83.5%)	14 (73.7%)	
Skin infiltration						0.004
Negative	36 (73.5%)	270 (87.1%)		270 (87.1%)	12 (63.2%)	
Positive	13 (26.5%)	40 (12.9%)	0.012	40 (12.9%)	7 (36.8%)	
Lymph node status						0.751
Negative	17 (34.7%)	109 (35.2%)		109 (35.2%)	6 (31.6%)	
Positive	32 (65.3%)	201 (64.8%)	0.949	201 (64.8%)	13 (68.4%)	
Estrogen receptor						0.117
Negative	28 (57.1%)	187 (60.3%)		187 (60.3%)	8 (42.1%)	
Positive	21 (42.9%)	123 (39.7%)	0.673	123 (39.7%)	11 (57.9%)	
Progesterone receptor						0.043
Negative	35 (71.4%)	254 (81.9%)		254 (81.9%)	12 (63.2%)	
Positive	14 (28.6%)	56 (18.1%)	0.085	56 (18.1%)	7 (36.8%)	
HER2						0.982
Negative	31 (63.3%)	195 (62.9%)		195 (62.9%)	12 (63.2%)	
Positive	18 (36.7%)	115 (37.1%)	0.961	115 (37.1%)	7 (36.8%)	
Ki67						0.409
≤ 14%	11 (22.4%)	102 (32.9%)		102 (32.9%)	8 (42.1%)	
> 14%	38 (77.6%)	208 (67.1%)	0.143	208 (67.1%)	11 (57.9%)	
Intrinsic subtype HRBC						0.054
non-HRBC	28 (57.1%)	184 (59.4%)		184 (59.4%)	7 (36.8%)	
HRBC	21 (42.9%)	126 (40.6%)	0.770	126 (40.6%)	12 (63.2%)	
Intrinsic subtype HER2BC						0.688
non- HER2BC	38 (77.6%)	232 (74.8%)		232 (74.8%)	15 (78.9%)	
HER2BC	11 (22.4%)	78 (25.2%)	0.683	78 (25.2%)	4 (21.1%)	
Intrinsic subtype TNBC						0.098
non- TNBC	32 (65.3%)	204 (65.8%)		204 (65.8%)	16 (84.2%)	
TNBC	17 (34.7%)	106 (34.2%)	0.945	106 (34.2%)	3 (15.8%)	
Objective response rate						0.476
Non-Responders	8 (16.3%)	32 (10.3%)		32 (10.3%)	1 (5.3%)	
Responders	41 (83.7%)	278 (89.7%)	0.215	278 (89.7%)	18 (94.7%)	
Pathological response						0.103
Non-pCR	29 (59.2%)	205 (66.1%)		205 (66.1%)	16 (84.2%)	
pCR	20 (40.8%)	105 (33.9%)	0.343	105 (33.9%)	3 (15.8%)	
TILs						0.073
Low	32 (65.3%)	163 (52.6%)		163 (52.6%)	14 (73.7%)	
High	17 (34.7%)	147 (47.4%)	0.097	147 (47.4%)	5 (26.3%)	

HER: human epidermal growth factor receptor. HRBC: hormone receptor-positive breast cancer (ER+ and/or PgR+). HER2BC: human epidermal growth factor receptor 2-enriched breast cancer (ER-, PgR-, and HER2+). TNBC: triple negative breast cancer (ER-, PgR-, and HER2-). pCR: pathological complete response. TILs: tumor-infiltrating lymphocytes

Among patients with HER2BC, those who were UW tended to have a higher pCR rate than those in other BMI categories (*p* = 0.065), despite their tendency to have a lower TIL density (*p* = 0.070) **(Supplementary Table** 3**)**. Moreover, patients with HER2BC whose BMIs were > 25 kg/m^2^ had a higher frequency of lymph node metastasis than did those with BMIs < 25 kg/m^2^ (*p* = 0.039). No correlation was found between BMI and TILs, ORR, or pCR.

Finally, among patients with TNBC, those who were UW had significantly lower TILs (*p* = 0.035) and a significantly lower ORR (*p* = 0.003) than did non-UW counterparts **(**Table 3**)**; no significant difference was observed in the pCR rate (*p* = 0.602). When using a BMI cutoff of 25 kg/m^2^ or 30 kg/m^2^, the TIL density tended to be lower among patients with the lower BMIs than in those with the higher values (*p* = 0.077 and *p* = 0.100, respectively); however, no significant differences in ORR or pCR were observed.

**Table 3:** Difference in clinicopathological features due to body mass index categorized in TNBC

hba	Body mass index (kg/m2)		*p* value	Body mass index (kg/m2		*p* value	Body mass index (kg/m2)		*p* value
	≤ 18.5 (*n* = 17)	> 18.5 (*n* = 109)		≤ 25 (*n* = 98)	> 25 (*n* = 28)		≤ 30 (*n* = 123)	> 30 (*n* = 3)	
Age (years old)									0.258
≤ 60	11 (64.7%)	78 (71.6%)		67 (68.4%)	22 (78.6%)		86 (69.9%)	3 (100.0%)	
> 60	6 (35.3%)	31 (28.4%)	0.564	31 (31.6%)	6 (21.4%)	0.296	37 (30.1%)	0 (0.0%)	
Tumor size (mm)									0.433
≤ 20.0	4 (23.5%)	17 (15.6%)		18 (18.4%)	3 (10.7%)		20 (16.3%)	1 (33.3%)	
> 20.0	13 (76.5%)	92 (84.4%)	0.414	80 (81.6%)	25 (89.3%)	0.338	103 (83.7%)	2 (66.7%)	
Skin infiltration									0.519
Negative	13 (76.5%)	98 (89.9%)		88 (89.8%)	23 (82.1%)		108 (87.8%)	3 (100.0%)	
Positive	4 (23.5%)	11 (10.1%)	0.112	10 (10.2%)	5 (17.9%)	0.270	15 (12.2%)	0 (0.0%)	
Lymph node status									0.952
Negative	4 (23.5%)	36 (33.0%)		32 (32.7%)	8 (28.6%)		39 (31.7%)	1 (33.3%)	
Positive	13 (76.5%)	73 (67.0%)	0.434	66 (67.3%)	20 (71.4%)	0.682	84 (68.3%)	2 (66.7%)	
Ki67									0.695
≤ 14%	2 (11.8%)	28 (25.7%)		24 (24.5%)	6 (21.4%)		29 (23.6%)	1 (33.3%)	
> 14%	15 (88.2%)	81 (74.3%)	0.210	74 (75.5%)	22 (78.6%)	0.737	94 (76.4%)	2 (66.7%)	
Objective response rate									0.504
Non-Responders	6 (35.3%)	10 (9.2%)		14 (14.3%)	2 (7.1%)		16 (13.0%)	0 (0.0%)	
Responders	11 (64.7%)	99 (90.8%)	0.003	84 (85.7%)	26 (92.9%)	0.317	107 (87.0%)	3 (100.0%)	
Pathological response									0.777
Non-pCR	9 (52.9%)	65 (59.6%)		58 (59.2%)	16 (57.1%)		72 (58.5%)	2 (66.7%)	
pCR	8 (47.1%)	44 (40.4%)	0.602	40 (40.8%)	12 (42.9%)	0.847	51 (41.5%)	1 (33.3%)	
TILs									0.100
Low	12 (70.6%)	47 (43.1%)		50 (51.0%)	9 (32.1%)		59 (48.0%)	0 (73.7%)	
High	5 (29.4%)	62 (56.9%)	0.035	48 (49.0%)	19 (67.9%)	0.077	64 (52.0%)	3 (100.0%)	

*TNBC* triple negative breast cancer, *pCR* pathological complete response, *TILs* tumor-infiltrating lymphocytes

### Differences in prognosis due to pCR and TILs

**Fig. 2:**
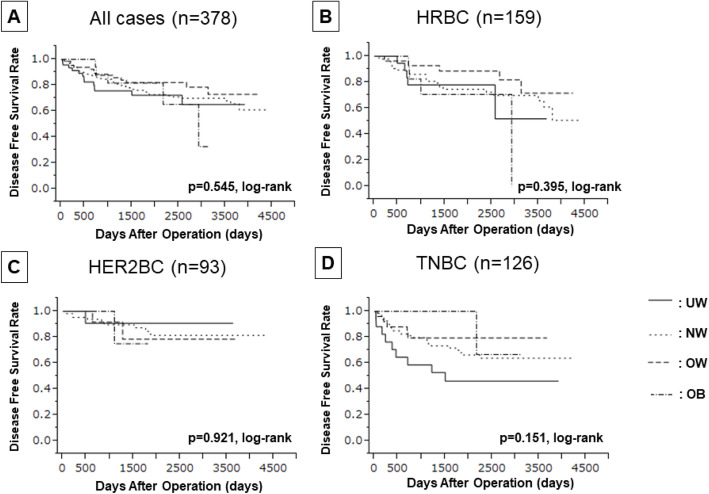
Kaplan-Meier stratification curve based on BMI categorized (UW; underweight, NW; normal weight, OW; overweight, OB; obese) for disease- free survival (DFS). **A** all case, **B** hormone receptor positive breast cancer (HRBC), **C** HER2-enriched breast cancer (HER2BC), **D** triple-negative breast cancer (TNBC)

**Fig. 3:**
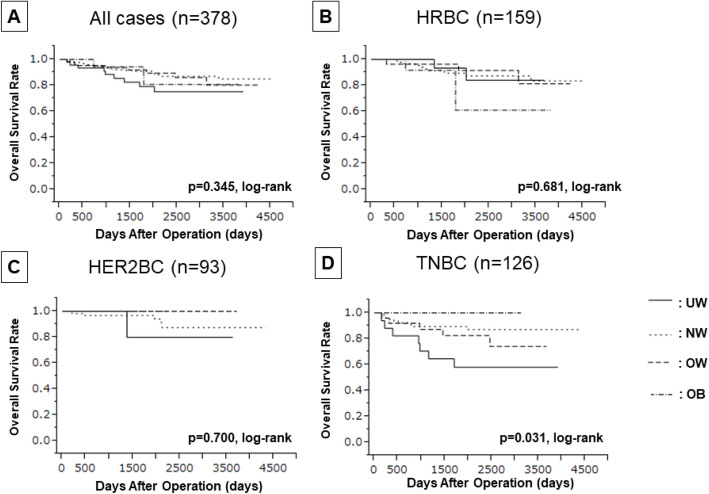
Kaplan-Meier stratification curve based on BMI categorized (UW; underweight, NW; normal weight, OW; overweight, OB; obese) for overall survival (OS). **A** all case, **B** hormone receptor positive breast cancer (HRBC), **C** HER2-enriched breast cancer (HER2BC), **D** triple-negative breast cancer (TNBC)

**Fig. 4:**
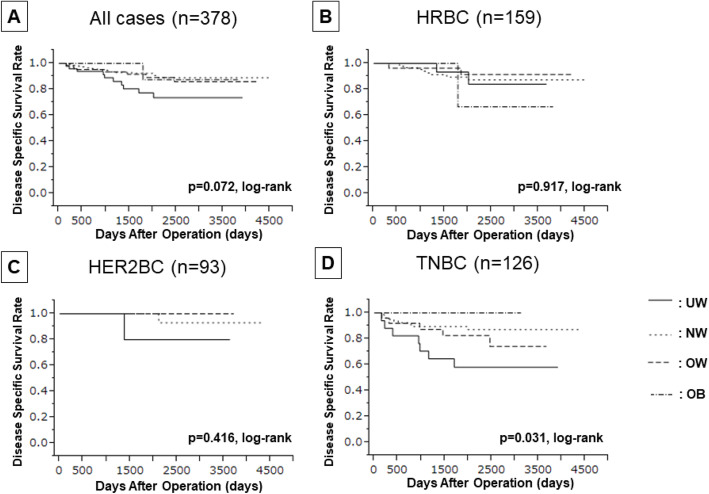
Kaplan-Meier stratification curve based on BMI categorized (UW; underweight, NW; normal weight, OW; overweight, OB; obese) for disease-specific survival (DFS). **A** all case, **B** hormone receptor positive breast cancer (HRBC), **C** HER2-enriched breast cancer (HER2BC), **D** triple-negative breast cancer (TNBC)

Differences in prognosis due to pCR and TILs were analyzed by Kaplan-Meier curve and log-rank test. In all cases, the pCR group showed a significant prolongation of DFS compared to the non-PCR group (log rank *p* < 0.001) **(Supplementaty Fig.** **2****)**. When divided into subtypes, this difference was found in HER2BC (log rank *p* = 0.013) and TNBC (log rank *p* = 0.001), but not in HRBC (log rank *p* = 0.243). In all cases, OS and DSS were significantly longer in the pCR group than in the non-PCR group (OS; log rank *p* = 0.008) **(Supplementaty Fig.** **3****)** (DSS; log rank *p* = 0.014) **(Supplementaty Fig.** **4****)**. In the prognostic analysis of OS and DSS by subgroup, the significant difference was observed only in TNBC (both OS and DSS; *p* = 0.001) **(Supplementaty Figs.** **3****,** **4****)**.

Next, prognosis analysis by Tils was performed. Regarding DFS, the results were similar to those examined by pCR, that is, the high TILs group showed a significant prolongation compared to the low TILs group in all case (log rank *p* = 0.002), HER2BC (log rank *p* = 0.012) and TNBC (log rank *p* = 0.007) **(Supplementaty Fig.** **5****)**. Regarding OS and DSS, no significant difference was observed in all cases (OS; log rank *p* = 0.194) **(Supplementaty Fig.** **6****)** (DSS; log rank *p* = 0.244) **(Supplementaty Fig.** **7****)**. However, when examined by subtype, TNBC showed a significant prolongation of OS and DSS in the high TILs group compared to the low TILs group (both OS and DSS; *p* = 0.028) **(Supplementaty Fig.** **6****,** **7****)**.

### Impact of BMI on prognosis

The DFS of all patients with breast cancer was analyzed with respect to their BMIs, but no significant difference was found (log rank *p* = 0.545) **(**Fig. 2**)**. Similar results were found when categorizing patients by their breast cancer types. On univariate analysis of DFS, no significant differences were found regardless of the BMI cut-off value (UW vs. NW/OW/OB: *p* = 0.191, HR = 0.680; UW/NW vs. OW/OB: *p* = 0.314, HR = 0.772; UW/NW/OW vs. OB: *p* = 0.435, HR = 1.395) **(Supplementary Table** **4****)**.

No significant differences in OS were found between patients with all types of cancer when categorized according to BMI (log rank *p* = 0.345). However, on univariate analysis, UW tended to be associated with a shorter OS (UW vs. NW/OW/OB: *p* = 0.055, HR = 0.476) (**Supplementary Table** **5**). Moreover, UW was associated with a significantly poorer DSS than the other BMI categories (UW vs. NW/OW/OB: *p* = 0.021, HR = 0.398) **(Supplementary Table** **6****)**.

Furthermore, when examining patients with each breast cancer type individually, the OS of those with TNBC who had low BMIs was significantly poorer than that of their high-BMI counterparts (log rank *p* = 0.031) **(**Fig. 3**)**. The cause of all deaths among patients with TNBC was breast cancer; as such, DSS data were identical (log rank p = 0.031) **(**Fig. 4**)**. Univariate analysis of DFS for patients with TNBC showed that those with UW tended to have poorer prognoses than did those of other BMI categories (UW vs. NW/OW/OB: *p* = 0.056, HR = 0.457). UW contributed to significantly shorter OS and DSS than did the other categories (UW vs. NW/OW/OB: *p* = 0.017, HR = 0.299) (Table 4). However, BMI was not an independent prognostic factor on multivariate analysis; moreover, no correlation between BMI and prognosis was found for patients with HER2BC specifically **(Supplementary Table** **7****)**.

**Table 4:** Univariate and multivariate analysis with respect to DFS, OS or DSS in TNBC

Disease-free survival						
	Univarite analysis			Multivariate analysis		
Parameters	Hazard ratio	95% CI	*p* value	Hazard ratio	95% CI	*p* value
Age at opetation (years old) ≤ 60 vs > 60	1.002	0.491–1.925	0.994			
Tumor size (mm) ≤ 20.0 vs > 20.0	0.924	0.434–2.277	0.850			
Skin infiltration Negative vs Positive	2.912	1.302–5.883	0.011	2.518	1.092–5.311	0.032
Lymph node status Negative vs Positive	1.466	0.741–3.160	0.281			
Ki67 ≤ 14% vs > 14%	1.413	0.684–3.294	0.367			
Objective response rate Non-Responders vs Responders	0.157	0.081–0.320	< 0.001	0.213	0.102–0.462	< 0.001
Pathological response Non-pCR vs pCR	0.286	0.122–0.590	< 0.001	0.427	0.176–0.929	0.031
TILs Low vs High	0.424	0.218–0.797	0.008	0.661	0.330–1.288	0.226
Body mass index (kg/m2) ≤ 18.5 vs > 18.5	0.457	0.227–1.022	0.056	0.604	0.290–1.377	0.218
Body mass index (kg/m2) ≤ 25.0 vs > 25.0	0.565	0.213–1.251	0.170			
Body mass index (kg/m2) ≤ 30.0 vs > 30.0	0.775	0.044–3.577	0.794			
Overall survival						
	Univarite analysis			Multivariate analysis		
Parameters	Hazard ratio	95% CI	*p* value	Hazard ratio	95% CI	*p* value
Age at opetation (years old) ≤ 60 vs > 60	0.722	0.236–1.844	0.514			
Tumor size (mm) ≤ 20.0 vs > 20.0	0.836	0.309–2.907	0.752			
Skin infiltration Negative vs Positive	4.423	1.675–10.643	0.004	3.957	1.395–10.667	0.011
Lymph node status Negative vs Positive	2.741	0.927–11.706	0.071	2.794	0.908–12.208	0.076
Ki67 ≤ 14% vs > 14%	2.090	0.707–8.928	0.199			
Objective response rate Non-Responders vs Responders	0.088	0.036–0.213	< 0.001	0.104	0.037–0.294	< 0.001
Pathological response Non-pCR vs pCR	0.129	0.020–0.446	< 0.001	0.246	0.038–0.913	0.035
TILs Low vs High	0.376	0.142–0.905	0.028	0.813	0.284–2.201	0.685
Body mass index (kg/m2) ≤ 18.5 vs > 18.5	0.299	0.124–0.788	0.017	0.645	0.248–1.832	0.393
Body mass index (kg/m2) ≤ 25.0 vs > 25.0	1.035	0.338–2.643	0.947			
Body mass index (kg/m2) ≤ 30.0 vs > 30.0	–	–	0.266			
Disease specific survival						
	Univarite analysis			Multivariate analysis		
Parameters	Hazard ratio	95% CI	*p* value	Hazard ratio	95% CI	*p* value
Age at opetation (years old) ≤ 60 vs > 60	0.722	0.236–1.844	0.514			
Tumor size (mm) ≤ 20.0 vs > 20.0	0.836	0.309–2.907	0.752			
Skin infiltration Negative vs Positive	4.423	1.675–10.643	0.004	3.957	1.395–10.667	0.011
Lymph node status Negative vs Positive	2.741	0.927–11.706	0.071	2.794	0.908–12.208	0.076
Ki67 ≤ 14% vs > 14%	2.090	0.707–8.928	0.199			
Objective response rate Non-Responders vs Responders	0.088	0.036–0.213	< 0.001	0.104	0.037–0.294	< 0.001
Pathological response Non-pCR vs pCR	0.129	0.020–0.446	< 0.001	0.246	0.038–0.913	0.035
TILs Low vs High	0.376	0.142–0.905	0.028	0.813	0.284–2.201	0.685
Body mass index (kg/m2) ≤ 18.5 vs > 18.5	0.299	0.124–0.788	0.017	0.645	0.248–1.832	0.393
Body mass index (kg/m2) ≤ 25.0 vs > 25.0	1.035	0.338–2.643	0.947			
Body mass index (kg/m2) ≤ 30.0 vs > 30.0	–	–	0.266			

*DFS* disease-free survival, *OS* overall survival, *DSS* disease specific survival, *TNBC* triple negative breast cancer, *CI* confidence intervals, *pCR* pathological complete response, *TILs* tumor-infiltrating lymphocytes

## Discussion

As mentioned above, obesity portends a poor prognosis for patients with breast cancer owing to the secretion of various hormones and cytokines, thereby causing chronic inflammatory conditions [5–10]. However, obesity was found not to be a poor prognosis factor in our study; in fact, OB patients with TNBC had improved prognoses. One explanation is that this study was performed at a single institution in East Asia; while the BMI distribution of our subjects was not markedly different from those in other studies from this part of the continent, OB patients are fewer in proportion than in Europe and the United States. Over 30% of patients with breast cancer are classified as OB in Europe and New Zealand [4, 11, 13, 35], compared to approximately 5% in East Asia [36–38]. One of the reasons that obesity is associated with poor prognosis among patients with breast cancer is suggested to be the lower rate of chemotherapy; for example, one study found that 20% of patients with breast cancer who had BMIs greater than 30 kg/m^2^ received reduced doses of chemotherapy [39]. It was also reported that OB patients with breast cancer have improved pCR rates and more favorable progression-free survival when they receive full (uncapped) doses of neoadjuvant chemotherapy [40]. Another study found that obesity was associated with a better prognosis among patients with hormone receptor-negative breast cancer but with a worse prognosis among those with HRBC [41]. These data support our own findings.

Conversely, being UW was associated with a poor prognosis among patients with TNBC. A number of studies from Europe and the United States investigated the relationship between UW and the prognosis of patients with breast cancer, although UW and NW were commonly considered a single group because of the relatively scarcity of the former [12–14]. The proportions of subjects with breast cancer in those geographic areas who were UW were 1–2% [11, 35]. However, UW patients with breast cancer are frequently found in Asia, and their clinicopathological features have been explored [37, 42–44]. These studies did not confirm the existence of associations between UW and prognosis owing to some inconsistencies between them. Depending on the study, UW patients were found to be younger [37, 43], have smaller tumors [37, 42, 43], rarely have lymph node metastases [37, 42], and have lower histologic grades [37]; one study showed them to have more frequent HER2 positivity [44], another found them to have more frequent hormone receptor positivity [37], and two others found them to be more frequently hormone receptor-negative [42, 43]. Regarding prognosis, a number of studies, including one pooled analysis, demonstrated poor survival outcomes not only in OB patients with breast cancer but also in UW patients [11, 37–39, 43–50]. However, there were difference in each subtype; some studies found that UW patients with HRBC had poorer prognoses [47–49] and that those with HER2BC or TNBC did not [47, 49]. One study found that UW patients with HER2BC had poor prognoses [37]. In contrast, our study found that UW was associated with a poor prognosis only in patients with TNBC.

The cause of poor prognosis among UW patients with breast cancer has been speculated on for some time. For example, one group found that UW is associated with a higher frequency of tumor progression [38]; however, such progression was not marked in other studies (including ours). Another study found that chemotherapy was frequently incomplete in UW patients [41], but our data suggested that this did not cause poorer prognosis because there were only a few patients who were unable to complete chemotherapy in our study. Some investigators cited the more aggressive breast cancer characteristics among younger patients, who comprised a large proportion of UW subjects, as a reason for poor prognosis [37, 43, 46, 47]; however, this was also not supported by our data. Others posited that the immune system is dysfunctional in UW individuals owing to chronic malnutrition and micronutrient deficiency [43, 51, 52]. Additionally, the effects of inflammatory reactions accompanied by cytokine expression and systemic immune reactions were considered [37, 48]; however, such studies did not identify the causal relationship between poor prognoses of patients with breast cancer and UW. Our study was able to demonstrate that the tumor microenvironment in UW patients may be more tumor-permissive owing to the low TIL density.

There are still few reports examining the correlation between BMI and TILs. In a report examining only TNBC, no significant correlation was found between BMI and TILs, but it was found that the prognostic effect for higher TILs weakened in obese patients [53]. One previous report reported that CD3 + lymphocytes were significantly reduced in obese breast cancer patients, affecting the therapeutic effect of immunotherapy [54]. The report further shows that obesity reduces cytotoxic T lymphocytes and increases macrophages and PD-1-positive lymphocytes that promote tumor growth with in vivo studies in mice. Other studies also reported that obesity caused changes in tumor-related macrophages, creating a tumor microenvironment in which tumors tend to grow [55, 56]. In other words, obesity may be more influenced by changes in the proportion of subtypes than changes in the density of TILs. In this study, TILs were examined using HE-stained specimens. As previously reported, it is judged that a subset analysis of TILs by immunohistochemical staining is also necessary. Beyond that, it may be necessary to analyze gene expression in the cancer itself and the cancerous stromal part. Elucidation of these is our task. However, in this study, not only TNBC but also HRBC and HER2BC are mentioned, and the therapeutic effect of the same regimen is examined, so it is judged to be valuable.

The largest limitation in our study was the small number of OB and UW patients with breast cancer. Post-diagnosis weight gain is also known to increase the risk of breast cancer recurrence [1, 57, 58], but we did not examine changes in body weight over time. Additionally, there may have been some BMI-associated confounding factors such as age, given that the frequency of UW patients with breast cancer was high among younger subjects [37, 43]. It has been reported that TILs are lower among the elderly than among younger individuals [59]; therefore, the composition of the immune microenvironment may change with age. No correlation was found between BMI and age in this study, although this should be considered with caution. Comorbidities, smoking, alcohol, and physical activity can also influence BMI, but these factors were not investigated in our study. However, in recent years, immunotherapy has been clinically used for various carcinomas. Among them, there are some reports that the therapeutic effect is high in obese patients [60–62]. It is also reported that the relationship between BMI and therapeutic effect is contradictory between cell-mediated antineoplastic agents and immunotherapy [61]. As a consideration of the cause, there is a report that considers the exhaustion of immunity associated with chronic inflammation due to obesity [60]. There is also a report that the expression of PD-1 of TILs is increased by increasing BMI [54], and the involvement of clinically used immunotherapy is deeply suspected.

## Conclusion

Our data did not support the hypothesis that obesity affects the tumor immune microenvironment; however, we showed that being UW does affect the tumor immune microenvironment.

## Supplementary Information

**Additional file 1 **: **Supplemental Fig. 1.** Classification by the tumor-infiltrating lymphocytes (TILs) density using hematoxylin and eosin-stained biopsy tissue. (A) > 50%, (B) > 10–50%, (C) ≤10%, and (D)absent.

**Additional file 2 **: **Supplemental Fig. 2.** Kaplan-Meier stratification curve based on pathological response for disease- free survival (DFS). (A) all case, (B) hormone receptor positive breast cancer (HRBC), (C) HER2-enriched breast cancer (HER2BC), (D) triple-negative breast cancer (TNBC).

**Additional file 3 **: **Supplemental Fig. 3.** Kaplan-Meier stratification curve based on pathological response for overall survival (OS). (A) all case, (B) hormone receptor positive breast cancer (HRBC), (C) HER2-enriched breast cancer (HER2BC), (D) triple-negative breast cancer (TNBC).

**Additional file 4 **: **Supplemental Fig. 4.** Kaplan-Meier stratification curve based on pathological response for disease-specific survival (DFS). (A) all case, (B) hormone receptor positive breast cancer (HRBC), (C) HER2-enriched breast cancer (HER2BC), (D) triple-negative breast cancer (TNBC).

**Additional file 5 **: **Supplemental Fig. 5.** Kaplan-Meier stratification curve based on tumor-infiltrating lymphocytes (TILs) for disease- free survival (DFS). (A) all case, (B) hormone receptor positive breast cancer (HRBC), (C) HER2-enriched breast cancer (HER2BC), (D) triple-negative breast cancer (TNBC).

**Additional file 6 **: **Supplemental Fig. 6.** Kaplan-Meier stratification curve based on tumor-infiltrating lymphocytes (TILs) for overall survival (OS). (A) all case, (B) hormone receptor positive breast cancer (HRBC), (C) HER2-enriched breast cancer (HER2BC), (D) triple-negative breast cancer (TNBC).

**Additional file 7 **: **Supplemental Fig. 7.** Kaplan-Meier stratification curve based on tumor-infiltrating lymphocytes (TILs) for disease-specific survival (DFS). (A) all case, (B) hormone receptor positive breast cancer (HRBC), (C) HER2-enriched breast cancer (HER2BC), (D) triple-negative breast cancer (TNBC).

**Additional file 8 **: **Supplementary Table 1.** Difference in clinicopathological features due to pathological response**. Supplementary Table 2.** Difference in clinicopathological features due to TILs.**. Supplementary Table 3.** Difference in clinicopathological features due to body mass index in HER2BC. **Supplementary Table 4.** Univariate and multivariate analysis with respect to disease-free survival. **Supplementary Table 5.** Univariate and multivariate analysis with respect to overall survival. **Supplementary Table 6.** Univariate and multivariate analysis with respect to disease specific survival. **Supplementary Table 7.** Univariate and multivariate analysis with respect to disease-free survival, overall survival or disease specific survival in HER2BC.

## Data Availability

The datasets used and/or analyzed during the current study are available from the corresponding author on reasonable request.

## Abbreviations

BMI: Body mass index
cCR: Clinical complete response
CIs: Confidence intervals
cPD: Clinical progressive disease
cPR: Clinical partial response
cSD: Clinical stable disease
CT: Computed tomography
DFS: Disease free survival
DSS: Disease specific survival
ER: Estrogen receptor
HER2: Human epidermal growth factor receptor 2
HER2BC: Human epidermal growth factor receptor 2-enriched breast cancer
HRBC: Hormone receptor positive breast cancer
HRs: Hazard ratios
NW: Normal weight
OB: Obese
ORR: Objective response rate
OS: Overall survival
OW: Overweight
pCR: Pathological complete response
PgR: Progesterone receptor
POC: Preoperative chemotherapy
TILs: Tumor-infiltrating lymphocytes
TNBC: Triple-negative breast cancer
US: Ultrasonography
UW: Underweight
WHO: World Health Organization
