# Supplementary figures and images for: Clinical verification of body mass index and tumor immune response in patients with breast cancer receiving preoperative chemotherapy
Source: BMC Cancer. 2021 Oct 20;21:1129. doi: 10.1186/s12885-021-08857-7 (PMC8529767; doi:10.1186/s12885-021-08857-7)

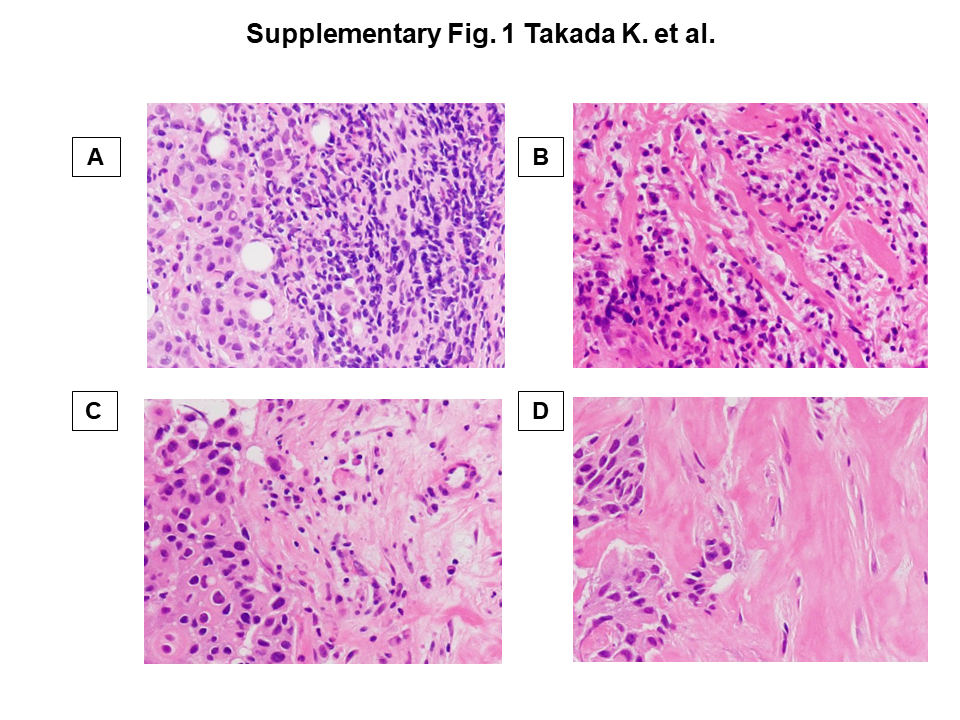

Supplement: Supplementary file 1 — Additional file 1 : Supplemental Fig. 1. Classification by the tumor-infiltrating lymphocytes (TILs) density using hematoxylin and eosin-stained biopsy tissue. (A) > 50%, (B) > 10–50%, (C) ≤10%, and (D)absent. [file 12885_2021_8857_MOESM1_ESM.tif]

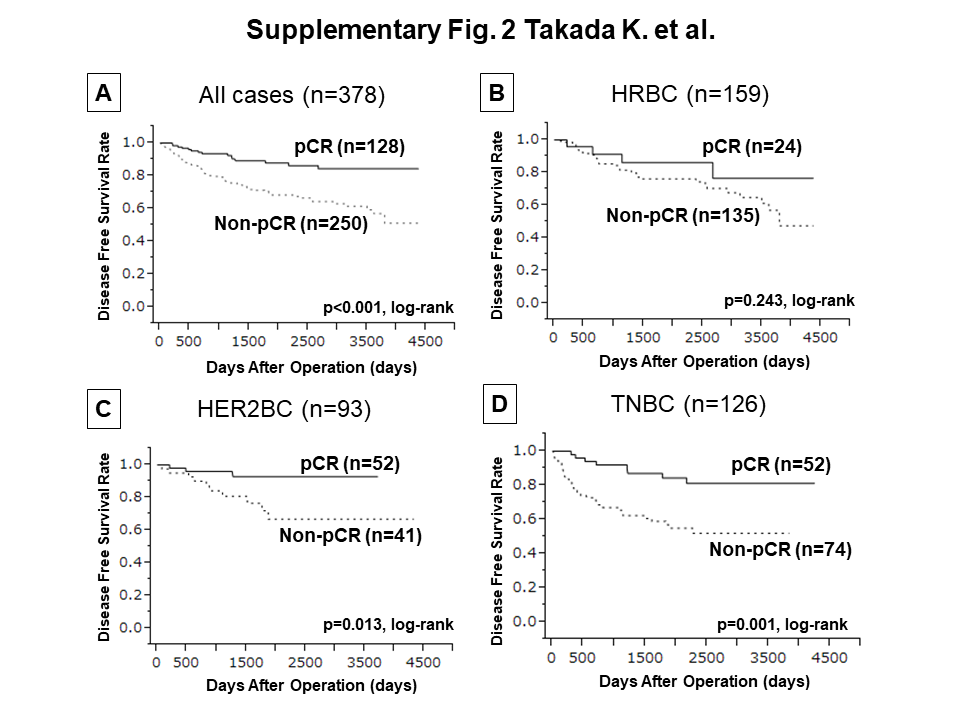

Supplement: Supplementary file 2 — Additional file 2 : Supplemental Fig. 2. Kaplan-Meier stratification curve based on pathological response for disease- free survival (DFS). (A) all case, (B) hormone receptor positive breast cancer (HRBC), (C) HER2-enriched breast cancer (HER2BC), (D) triple-negative breast cancer (TNBC). [file 12885_2021_8857_MOESM2_ESM.tif]

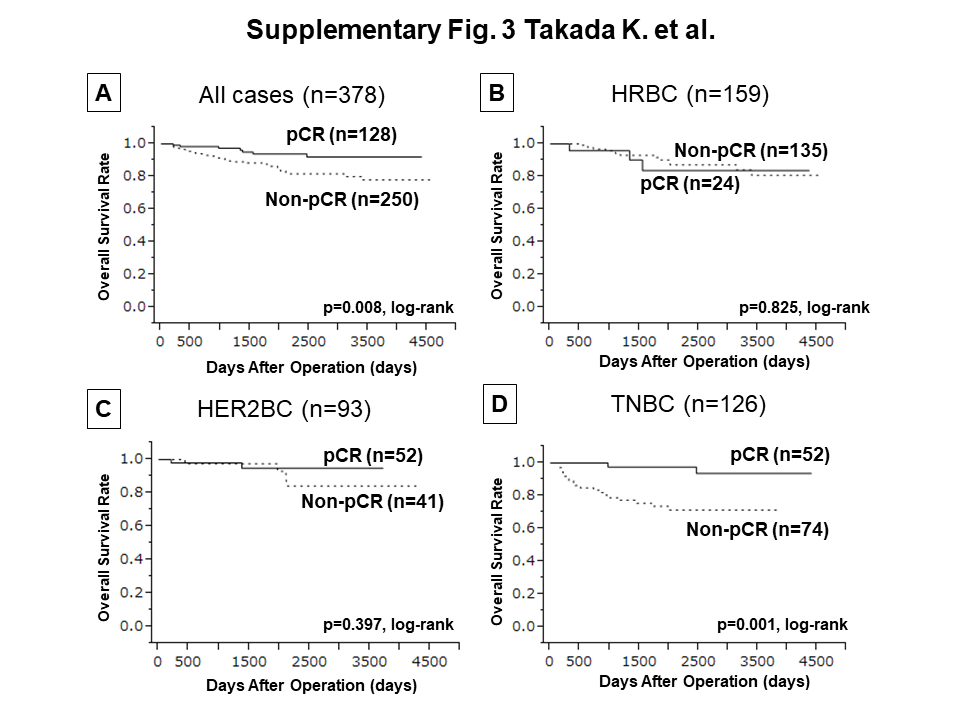

Supplement: Supplementary file 3 — Additional file 3 : Supplemental Fig. 3. Kaplan-Meier stratification curve based on pathological response for overall survival (OS). (A) all case, (B) hormone receptor positive breast cancer (HRBC), (C) HER2-enriched breast cancer (HER2BC), (D) triple-negative breast cancer (TNBC). [file 12885_2021_8857_MOESM3_ESM.tif]

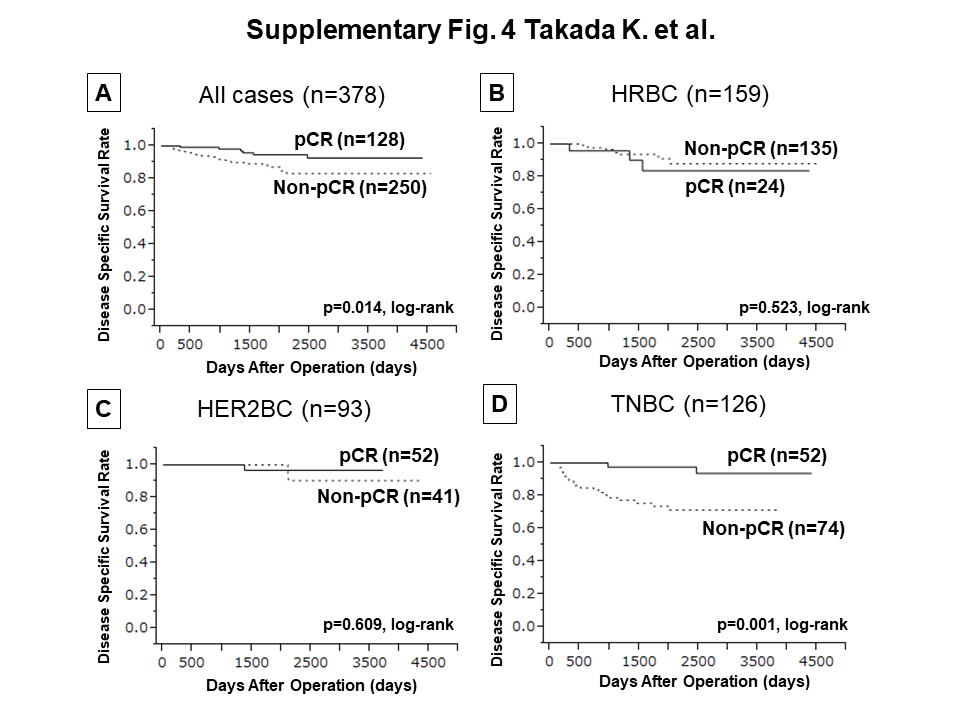

Supplement: Supplementary file 4 — Additional file 4 : Supplemental Fig. 4. Kaplan-Meier stratification curve based on pathological response for disease-specific survival (DFS). (A) all case, (B) hormone receptor positive breast cancer (HRBC), (C) HER2-enriched breast cancer (HER2BC), (D) triple-negative breast cancer (TNBC). [file 12885_2021_8857_MOESM4_ESM.tif]

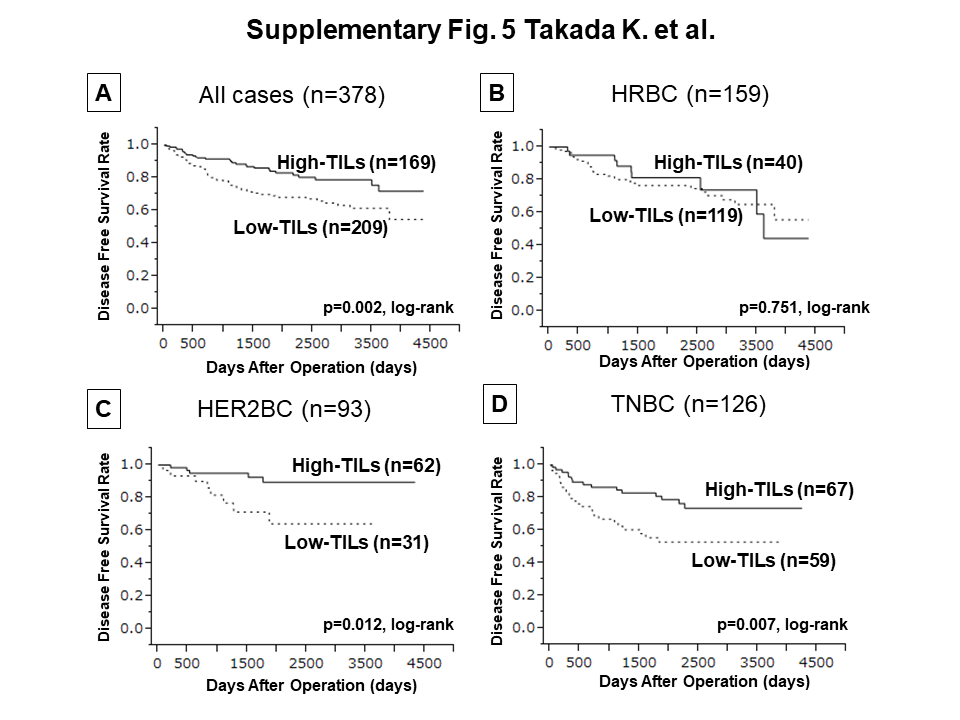

Supplement: Supplementary file 5 — Additional file 5 : Supplemental Fig. 5. Kaplan-Meier stratification curve based on tumor-infiltrating lymphocytes (TILs) for disease- free survival (DFS). (A) all case, (B) hormone receptor positive breast cancer (HRBC), (C) HER2-enriched breast cancer (HER2BC), (D) triple-negative breast cancer (TNBC). [file 12885_2021_8857_MOESM5_ESM.tif]

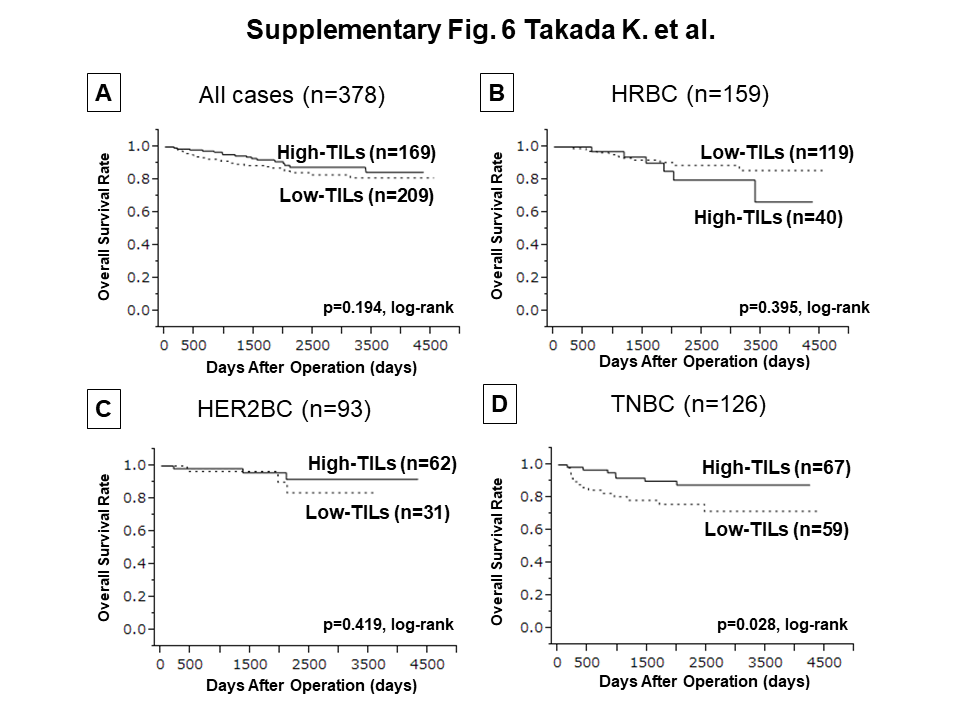

Supplement: Supplementary file 6 — Additional file 6 : Supplemental Fig. 6. Kaplan-Meier stratification curve based on tumor-infiltrating lymphocytes (TILs) for overall survival (OS). (A) all case, (B) hormone receptor positive breast cancer (HRBC), (C) HER2-enriched breast cancer (HER2BC), (D) triple-negative breast cancer (TNBC). [file 12885_2021_8857_MOESM6_ESM.tif]

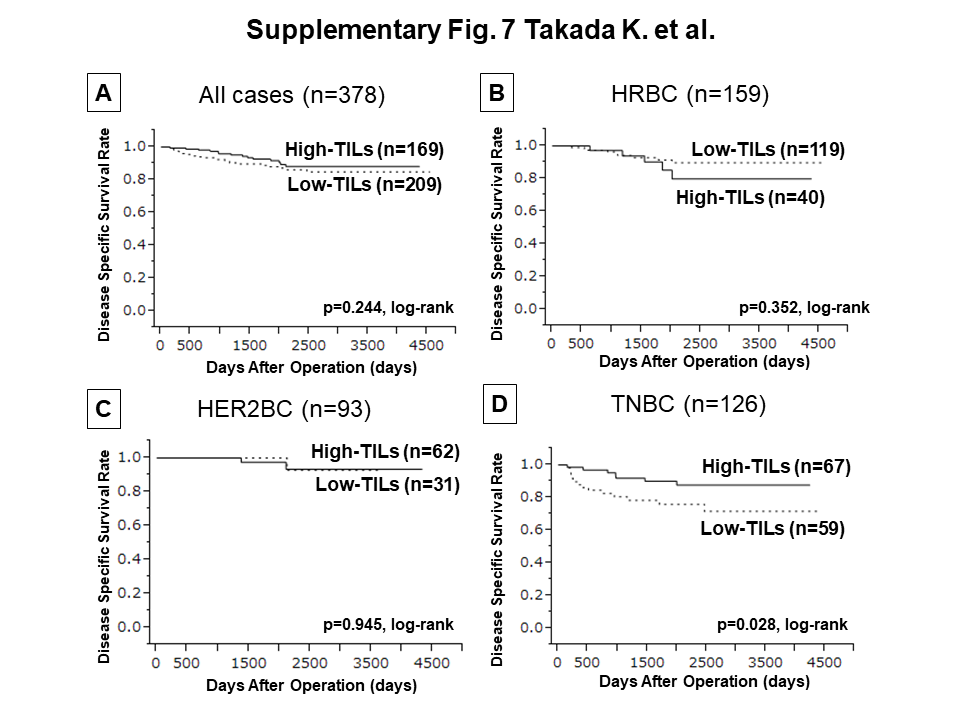

Supplement: Supplementary file 7 — Additional file 7 : Supplemental Fig. 7. Kaplan-Meier stratification curve based on tumor-infiltrating lymphocytes (TILs) for disease-specific survival (DFS). (A) all case, (B) hormone receptor positive breast cancer (HRBC), (C) HER2-enriched breast cancer (HER2BC), (D) triple-negative breast cancer (TNBC). [file 12885_2021_8857_MOESM7_ESM.tif]
