# Supplementary material for: Clinical verification of body mass index and tumor immune response in patients with breast cancer receiving preoperative chemotherapy
Source: BMC Cancer. 2021 Oct 20;21:1129. doi: 10.1186/s12885-021-08857-7 (PMC8529767; doi:10.1186/s12885-021-08857-7)
Supplement: Supplementary file 8 — Additional file 8 : Supplementary Table 1. Difference in clinicopathological features due to pathological response. Supplementary Table 2. Difference in clinicopathological features due to TILs.. Supplementary Table 3. Difference in clinicopathological features due to body mass index in HER2BC. Supplementary Table 4. Univariate and multivariate analysis with respect to disease-free survival. Supplementary Table 5. Univariate and multivariate analysis with respect to overall survival. Supplementary Table 6. Univariate and multivariate analysis with respect to disease specific survival. Supplementary Table 7. Univariate and multivariate analysis with respect to disease-free survival, overall survival or disease specific survival in HER2BC. [file 12885_2021_8857_MOESM8_ESM.docx]

**Supplementary Table 1.** **Difference in clinicopathological features due to TILs**

| Parameters | All case (*n* = 378) | | | TNBC (*n* =126) | | | HER2BC (*n* =93) | | |
| --- | --- | --- | --- | --- | --- | --- | --- | --- | --- |
|  | TILs | | *p* value | TILs | | *p* value | TILs | | *p* value |
|  | Low (*n* =209) | High (*n* =169) |  | Low (*n* =59) | High (*n* =67) |  | Low (*n* =31) | High (*n* =62) |  |
| Age (years old)  ≤ 60  > 60 | 139 (66.5%)  70 (33.5%) | 116 (68.6%)  53 (31.4%) | 0.660 | 38 (64.4%)  21 (35.6%) | 51 (76.1%)  16 (23.9%) | 0.150 | 18 (58.1%)  13 (41.9%) | 34 (54.8%)  28 (45.2%) | 0.768 |
| Tumor size (mm)  ≤ 20.0  > 20.0 | 38 (18.2%)  171 (81.8%) | 27 (16.0%)  142 (84.0%) | 0.572 | 10 (16.9%)  49 (83.1%) | 11 (16.4%)  56 (83.6%) | 0.936 | 4 (12.9%)  27 (87.1%) | 12 (19.4%)  50 (80.6%) | 0.437 |
| Skin infiltration  Negative  Positive | 166 (79.4%)  43 (20.6%) | 152 (89.9%)  17 (10.1%) | 0.005 | 49 (83.1%)  10 (16.9%) | 62 (92.5%)  5 (7.5%) | 0.101 | 22 (71.0%)  9 (29.0%) | 55 (88.7%)  7 (11.3%) | 0.033 |
| Lymph node status  Negative  Positive | 69 (33.0%)  140 (67.0%) | 63 (37.3%)  106 (62.7%) | 0.387 | 19 (32.2%)  40 (67.8%) | 21 (31.3%)  46 (68.7%) | 0.918 | 11 (35.5%)  20 (64.5%) | 28 (45.2%)  34 (54.8%) | 0.373 |
| Estrogen receptor  Negative  Positive | 93 (44.5%)  116 (55.5%) | 130 (76.9%)  39 (23.1%) | <0.001 | -  - | -  - | - | -  - | -  - | - |
| Progesterone receptor  Negative  Positive | 148 (70.8%)  61 (29.2%) | 153 (90.5%)  16 (9.5%) | <0.001 | -  - | -  - | - | -  - | -  - | - |
| HER2  Negative  Positive | 143 (68.4%)  66 (31.6%) | 95 (56.2%)  74 (43.8%) | 0.015 | -  - | -  - | - | -  - | -  - | - |
| Ki67  ≤14 %  >14 % | 86 (41.1%)  123 (58.9%) | 35 (20.7%)  134 (79.3%) | <0.001 | 21 (35.6%)  38 (64.4%) | 9 (13.4%)  58 (86.6%) | 0.004 | 11 (35.5%)  20 (64.5%) | 15 (24.2%)  47 (75.8%) | 0.253 |
| Intrinsic subtype HRBC  non-HRBC  HRBC | 90 (43.1%)  119 (56.9%) | 129 (76.3%)  40 (23.7%) | <0.001 | -  - | -  - | - | -  - | -  - | - |
| Intrinsic subtype HER2BC  non- HER2BC  HER2BC | 178 (85.2%)  31 (14.8%) | 107 (63.3%)  62 (36.7%) | <0.001 | -  - | -  - | - | -  - | -  - | - |
| Intrinsic subtype TNBC  non- TNBC  TNBC | 150 (71.8%)  59 (28.2%) | 102 (60.4%)  67 (39.6%) | 0.019 | -  - | -  - | - | -  - | -  - | - |
| Objective response rate  Non-Responders  Responders | 33 (15.8%)  176 (84.2%) | 8 (4.7%)  161 (95.3%) | 0.001 | 12 (20.3%)  47 (79.7%) | 4 (6.0%)  63 (94.0%) | 0.016 | 4 (12.9%)  27 (87.1%) | 1 (1.6%)  61 (98.4%) | 0.023 |
| Pathological response  Non-pCR  pCR | 165 (78.9%)  44 (21.1%) | 85 (50.3%)  84 (49.7%) | <0.001 | 42 (71.2%)  17 (28.8%) | 32 (47.8%)  35 (52.2%) | 0.008 | 19 (61.3%)  12 (38.7%) | 22 (35.5%)  40 (64.5%) | 0.018 |
| Body mass index (kg/m2)  ≤18.5  >18.5 | 32 (15.3%)  177 (84.7%) | 17 (10.1%)  152 (89.9%) | 0.131 | 12 (20.3%)  47 (79.7%) | 5 (7.5%)  62 (92.5%) | 0.035 | 5 (16.1%)  26 (83.9%) | 6 (9.7%)  56 (90.3%) | 0.364 |
| Body mass index (kg/m2)  ≤25  >25 | 159 (76.1%)  50 (23.9%) | 134 (79.3%)  35 (20.7%) | 0.457 | 50 (84.7%)  9 (15.3%) | 48 (71.6%)  19 (28.4%) | 0.077 | 23 (74.2%)  8 (25.8%) | 54 (87.1%)  8 (12.9%) | 0.120 |
| Body mass index (kg/m2)  ≤30  >30 | 195 (93.3%)  14 (6.7%) | 164 (97.0%)  5 (3.0%) | 0.098 | 59 (100.0%)  0 (0.0%) | 64 (95.5%)  3 (4.5%) | 0.100 | 29 (93.5%)  2 (6.5%) | 60 (96.8%)  2 (3.2%) | 0.470 |

TILs: tumor-infiltrating lymphocytes. TNBC: triple negative breast cancer (ER-, PgR-, and HER2-). HER2BC: human epidermal growth factor receptor 2-enriched breast cancer (ER-, PgR-, and HER2+). HRBC: hormone receptor-positive breast cancer (ER+ and/or PgR+). pCR: pathological complete response.

**Supplementary Table 2. Difference in clinicopathological features due to body mass index in HER2BC**

|  | Body mass index (kg/m2) | | *p* value | Body mass index (kg/m2) | | *p* value | Body mass index (kg/m2) | | *p* value |
| --- | --- | --- | --- | --- | --- | --- | --- | --- | --- |
|  | ≤ 18.5  (*n* =11) | 18.5<  (*n* =82) |  | ≤ 25  (*n* =77) | 25<  (*n* =16) |  | ≤ 30  (*n* =89) | 30<  (*n* =4) |  |
| Age (years old)  ≤ 60  > 60 | 4 (36.4%)  7 (63.6%) | 48 (58.5%)  34 (41.5%) | 0.164 | 43 (55.8%)  34 (44.2%) | 9 (56.3%)  7 (43.8%) | 0.976 | 50 (56.2%)  39 (43.8%) | 2 (50.0%)  2 (50.0%) | 0.808 |
| Tumor size (mm)  ≤ 20.0  > 20.0 | 2 (18.2%)  9 (81.8%) | 14 (17.1%)  68 (82.9%) | 0.927 | 15 (19.5%)  62 (80.5%) | 1 (6.2%)  15 (93.8%) | 0.202 | 16 (18.0%)  73 (82.0%) | 0 (0.0%)  4 (100.0%) | 0.351 |
| Skin infiltration  Negative  Positive | 8 (72.7%)  3 (27.3%) | 69 (84.1%)  13 (15.9%) | 0.346 | 65 (84.4%)  12 (15.6%) | 12 (75.0%)  4 (25.0%) | 0.364 | 75 (84.3%)  14 (15.7%) | 2 (50.0%)  2 (50.0%) | 0.076 |
| Lymph node status  Negative  Positive | 6 (54.5%)  5 (45.5%) | 33 (40.2%)  49 (59.8%) | 0.367 | 36 (46.8%)  41 (53.2%) | 3 (18.8%)  13 (81.3%) | 0.039 | 39 (43.8%)  50 (56.2%) | 0 (0.0%)  4 (100.0%) | 0.082 |
| Ki67  ≤14 %  >14 % | 1 (9.1%)  10 (90.9%) | 25 (30.5%)  57 (69.5%) | 0.138 | 23 (29.9%)  54 (70.1%) | 3 (18.8%)  13 (81.3%) | 0.367 | 25 (28.1%)  64 (71.9%) | 1 (25.0%)  3 (75.0%) | 0.893 |
| Objective response rate  Non-Responders  Responders | 0 (0.0%)  11 (100.0%) | 5 (6.1%)  77 (93.9%) | 0.400 | 3 (3.9%)  74 (96.1%) | 2 (12.5%)  14 (87.5%) | 0.165 | 4 (4.5%)  85 (95.5%) | 1 (25.0%)  3 (75.0%) | 0.075 |
| Pathological response  Non-pCR  pCR | 2 (18.2%)  9 (81.8%) | 39 (47.6%)  43 (52.4%) | 0.065 | 31 (40.3%)  46 (59.7%) | 10 (62.5%)  6 (37.5%) | 0.103 | 38 (42.7%)  51 (57.3%) | 3 (75.0%)  1 (25.0%) | 0.203 |
| TILs  Low  High | 5 (45.5%)  6 (54.5%) | 26 (31.7%)  56 (68.3%) | 0.070 | 23 (29.9%)  54 (70.1%) | 8 (50.0%)  8 (50.0%) | 0.120 | 29 (32.6%)  60 (67.4%) | 2 (50.0%)  2 (50.0%) | 0.470 |

HER2BC: human epidermal growth factor receptor 2-enriched breast cancer (ER-, PgR-, and HER2+). pCR: pathological complete response. TILs: tumor-infiltrating lymphocytes.

**Supplementary Table 3.** **Univariate and multivariate analysis with respect to disease-free survival**

|  | Univarite analysis | | |  | Multivariate analysis | | |
| --- | --- | --- | --- | --- | --- | --- | --- |
| Parameters | Hazard ratio | 95% CI | *p* value |  | Hazard ratio | 95% CI | *p* value |
| Age at opetation (yr)  ≤ 60 vs > 60 | 0.865 | 0.544-1.338 | 0.522 |  |  |  |  |
| Tumor size (mm)  ≤ 20.0 vs > 20.0 | 1.359 | 0.771-2.622 | 0.304 |  |  |  |  |
| Skin infiltration  Negative vs Positive | 1.769 | 1.048-2.849 | 0.033 |  | 1.415 | 0.816-2.355 | 0.209 |
| Lymph node status  Negative vs Positive | 2.149 | 1.131-3.708 | 0.002 |  | 2.014 | 1.219-3.504 | 0.006 |
| Estrogen receptor  Negative vs Positive | 0.913 | 0.598-1.377 | 0.667 |  |  |  |  |
| Progesterone receptor  Negative vs Positive | 0.728 | 0.416-1.203 | 0.225 |  |  |  |  |
| HER2  Negative vs Positive | 0.464 | 0.272-0.751 | 0.001 |  | 0.547 | 0.206-1.217 | 0.147 |
| Ki67  ≤14 % vs >14 % | 1.265 | 0.821-2.002 | 0.292 |  |  |  |  |
| Intrinsic subtype HRBC  non-HRBC vs HRBC | 0.997 | 0.658-1.500 | 0.990 |  |  |  |  |
| Intrinsic subtype HER2BC  non- HER2BC vs HER2BC | 0.501 | 0.266-0.869 | 0.012 |  | 1.632 | 0.627-4.753 | 0.323 |
| Intrinsic subtype TNBC  non- TNBC vs TNBC | 1.571 | 1.036-2.364 | 0.034 |  | 1.928 | 1.184-3.147 | 0.008 |
| Objective response rate  Non-Responders vs Responders | 0.326 | 0.204-0.545 | <0.001 |  | 0.375 | 0.227-0.642 | 0.001 |
| Pathological response  Non-pCR vs pCR | 0.335 | 0.185-0.565 | <0.001 |  | 0.427 | 0.228-0.754 | 0.003 |
| TILs  Low vs High | 0.504 | 0.320-0.774 | 0.002 |  | 0.637 | 0.390-1.023 | 0.062 |
| Body mass index (kg/m2)  ≤ 18.5 vs > 18.5 | 0.680 | 0.403-1.229 | 0.191 |  |  |  |  |
| Body mass index (kg/m2)  ≤ 25.0 vs > 25.0 | 0.772 | 0.448-1.262 | 0.314 |  |  |  |  |
| Body mass index (kg/m2)  ≤ 30.0 vs > 30.0 | 1.395 | 0.542-2.937 | 0.453 |  |  |  |  |

CI: confidence intervals. HER: human epidermal growth factor receptor. HRBC: hormone receptor-positive breast cancer (ER+ and/or PgR+). HER2BC: human epidermal growth factor receptor 2-enriched breast cancer (ER-, PgR-, and HER2+). TNBC: triple negative breast cancer (ER-, PgR-, and HER2-). pCR: pathological complete response. TILs: tumor-infiltrating lymphocytes.

**Supplementary Table 4. Univariate and multivariate analysis with respect to overall survival**

|  | Univarite analysis | | |  | Multivariate analysis | | |
| --- | --- | --- | --- | --- | --- | --- | --- |
| Parameters | Hazard ratio | 95% CI | *p* value |  | Hazard ratio | 95% CI | *p* value |
| Age at opetation (yr)  ≤ 60 vs > 60 | 0.724 | 0.349-1.388 | 0.342 |  |  |  |  |
| Tumor size (mm)  ≤ 20.0 vs > 20.0 | 1.511 | 0.654-4.385 | 0.360 |  |  |  |  |
| Skin infiltration  Negative vs Positive | 2.916 | 1.497-5.409 | 0.002 |  | 2.772 | 1.365-5.411 | 0.006 |
| Lymph node status  Negative vs Positive | 2.935 | 1.335-7.738 | 0.006 |  | 2.602 | 1.172-6.905 | 0.017 |
| Estrogen receptor  Negative vs Positive | 0.742 | 0.387-1.363 | 0.342 |  |  |  |  |
| Progesterone receptor  Negative vs Positive | 0.533 | 0.202-1.170 | 0.123 |  |  |  |  |
| HER2  Negative vs Positive | 0.303 | 0.115-0.665 | 0.002 |  | - | - | 0.002 |
| Ki67  ≤14 % vs >14 % | 1.186 | 0.640-2.306 | 0.596 |  |  |  |  |
| Intrinsic subtype HRBC  non-HRBC vs HRBC | 0.855 | 0.457-1.554 | 0.611 |  |  |  |  |
| Intrinsic subtype HER2BC  non- HER2BC vs HER2BC | 0.501 | 0.190-1.100 | 0.088 |  | - | - | 0.020 |
| Intrinsic subtype TNBC  non- TNBC vs TNBC | 1.802 | 0.990-3.263 | 0.054 |  | 1.644 | 0.849-3.216 | 0.140 |
| Objective response rate  Non-Responders vs Responders | 0.251 | 0.134-0.498 | <0.001 |  | 0.230 | 0.116-0.476 | <0.001 |
| Pathological response  Non-pCR vs pCR | 0.353 | 0.144-0.744 | 0.005 |  | 0.484 | 0.188-1.098 | 0.084 |
| TILs  Low vs High | 0.667 | 0.353-1.218 | 0.190 |  |  |  |  |
| Body mass index (kg/m2)  ≤ 18.5 vs > 18.5 | 0.476 | 0.244-1.017 | 0.055 |  | 0.593 | 0.299-1.286 | 0.176 |
| Body mass index (kg/m2)  ≤ 25.0 vs > 25.0 | 0.963 | 0.451-1.879 | 0.917 |  |  |  |  |
| Body mass index (kg/m2)  ≤ 30.0 vs > 30.0 | 0.996 | 0.162-3.240 | 0.995 |  |  |  |  |

CI: confidence intervals. HER: human epidermal growth factor receptor. HRBC: hormone receptor-positive breast cancer (ER+ and/or PgR+). HER2BC: human epidermal growth factor receptor 2-enriched breast cancer (ER-, PgR-, and HER2+). TNBC: triple negative breast cancer (ER-, PgR-, and HER2-). pCR: pathological complete response. TILs: tumor-infiltrating lymphocytes.

**Supplementary Table 5. Univariate and multivariate analysis with respect to disease specific survival**

|  | Univarite analysis | | |  | Multivariate analysis | | |
| --- | --- | --- | --- | --- | --- | --- | --- |
| Parameters | Hazard ratio | 95% CI | *p* value |  | Hazard ratio | 95% CI | *p* value |
| Age at opetation (yr)  ≤ 60 vs > 60 | 0.669 | 0.298-1.357 | 0.276 |  |  |  |  |
| Tumor size (mm)  ≤ 20.0 vs > 20.0 | 1.263 | 0.539-3.691 | 0.617 |  |  |  |  |
| Skin infiltration  Negative vs Positive | 2.476 | 1.176-4.862 | 0.019 |  | 2.572 | 1.168-5.352 | 0.020 |
| Lymph node status  Negative vs Positive | 4.032 | 1.606-13.517 | 0.002 |  | 3.625 | 1.429-12.223 | 0.005 |
| Estrogen receptor  Negative vs Positive | 0.668 | 0.325-1.296 | 0.238 |  |  |  |  |
| Progesterone receptor  Negative vs Positive | 0.654 | 0.246-1.455 | 0.316 |  |  |  |  |
| HER2  Negative vs Positive | 0.163 | 0.039-0.452 | <0.001 |  | - | - | 0.003 |
| Ki67  ≤14 % vs >14 % | 1.356 | 0691-2.855 | 0.386 |  |  |  |  |
| Intrinsic subtype HRBC  non-HRBC vs HRBC | 0.801 | 0.404-1.529 | 0.506 |  |  |  |  |
| Intrinsic subtype HER2BC  non- HER2BC vs HER2BC | 0.270 | 0.065-0.749 | 0.009 |  | - | - | 0.093 |
| Intrinsic subtype TNBC  non- TNBC vs TNBC | 2.425 | 1.281-4.657 | 0.007 |  | 1.979 | 0.989-4.058 | 0.054 |
| Objective response rate  Non-Responders vs Responders | 0.231 | 0.119-0.476 | <0.001 |  | 0.207 | 0.099-0.451 | <0.001 |
| Pathological response  Non-pCR vs pCR | 0.351 | 0.132-0.781 | 0.009 |  | 0.484 | 0.174-1.162 | 0.108 |
| TILs  Low vs High | 0.678 | 0.342-1.293 | 0.241 |  |  |  |  |
| Body mass index (kg/m2)  ≤ 18.5 vs > 18.5 | 0.398 | 0.200-0.861 | 0.021 |  | 0.520 | 0.255-1.145 | 0.100 |
| Body mass index (kg/m2)  ≤ 25.0 vs > 25.0 | 0.864 | 0.369-1.796 | 0.710 |  |  |  |  |
| Body mass index (kg/m2)  ≤ 30.0 vs > 30.0 | 0.548 | 0.031-2.531 | 0.513 |  |  |  |  |

CI: confidence intervals. HER: human epidermal growth factor receptor. HRBC: hormone receptor-positive breast cancer (ER+ and/or PgR+). HER2BC: human epidermal growth factor receptor 2-enriched breast cancer (ER-, PgR-, and HER2+). TNBC: triple negative breast cancer (ER-, PgR-, and HER2-). pCR: pathological complete response. TILs: tumor-infiltrating lymphocytes.

**Supplementary Table 6. Univariate and multivariate analysis with respect to disease-free survival, overall survival or disease specific survival in HER2BC**

| Disease-free survival | | | | | | | | | | | | | | | |
| --- | --- | --- | --- | --- | --- | --- | --- | --- | --- | --- | --- | --- | --- | --- | --- |
|  | | Univarite analysis | | | | | |  | | Multivariate analysis | | | | | |
| Parameters | | Hazard ratio | | 95% CI | | *p* value | |  | | Hazard ratio | | 95% CI | | *p* value | |
| Age at opetation (yr)  ≤ 60 vs > 60 | | 0.367 | | 0.082-1.200 | | 0.101 | |  | |  | |  | |  | |
| Tumor size (mm)  ≤ 20.0 vs > 20.0 | | 1.569 | | 0.301-28.792 | | 0.648 | |  | |  | |  | |  | |
| Skin infiltration  Negative vs Positive | | 1.420 | | 0.318-4.645 | | 0.607 | |  | |  | |  | |  | |
| Lymph node status  Negative vs Positive | | 2.197 | | 0.671-9.811 | | 0.204 | |  | |  | |  | |  | |
| Ki67  ≤14 % vs >14 % | | 1.072 | | 0.348-3.972 | | 0.907 | |  | |  | |  | |  | |
| Objective response rate  Non-Responders vs Responders | | 0.121 | | 0.038-0.453 | | 0.003 | |  | | 0.275 | | 0.066-1.183 | | 0.080 | |
| Pathological response  Non-pCR vs pCR | | 0.225 | | 0.050-0.737 | | 0.013 | |  | | 0.361 | | 0.075-1.373 | | 0.137 | |
| TILs  Low vs High | | 0.262 | | 0.079-0.789 | | 0.018 | |  | | 0.511 | | 0.134-2.024 | | 0.325 | |
| Body mass index (kg/m2)  ≤ 18.5 vs > 18.5 | | 1.385 | | 0.272-25.250 | | 0.744 | |  | |  | |  | |  | |
| Body mass index (kg/m2)  ≤ 25.0 vs > 25.0 | | 1.401 | | 0.313-4.599 | | 0.620 | |  | |  | |  | |  | |
| Body mass index (kg/m2)  ≤ 30.0 vs > 30.0 | | 1.861 | | 0.102-9.635 | | 0.586 | |  | |  | |  | |  | |
| Overall survival | | | | | | | | | | | | | | | |
|  | | Univarite analysis | | | | | |  | | Multivariate analysis | | | | | |
| Parameters | | Hazard ratio | | 95% CI | | *p* value | |  | | Hazard ratio | | 95% CI | | *p* value | |
| Age at opetation (yr)  ≤ 60 vs > 60 | | 0.279 | | 0.015-1.732 | | 0.187 | |  | |  | |  | |  | |
| Tumor size (mm)  ≤ 20.0 vs > 20.0 | | - | | - | | 0.276 | |  | |  | |  | |  | |
| Skin infiltration  Negative vs Positive | | 2.321 | | 0.322-11.903 | | 0.359 | |  | |  | |  | |  | |
| Lymph node status  Negative vs Positive | | 2.805 | | 0.449-53.855 | | 0.298 | |  | |  | |  | |  | |
| Ki67  ≤14 % vs >14 % | | 0.245 | | 0.034-1.264 | | 0.093 | |  | | 0.281 | | 0.038-1.466 | | 0.132 | |
| Objective response rate  Non-Responders vs Responders | | 0.169 | | 0.033-1.224 | | 0.073 | |  | | 0.202 | | 0.039-1.478 | | 0.104 | |
| Pathological response  Non-pCR vs pCR | | 0.487 | | 0.067-2.506 | | 0.393 | |  | |  | |  | |  | |
| TILs  Low vs High | | 0.523 | | 0.096-2.829 | | 0.431 | |  | |  | |  | |  | |
| Body mass index (kg/m2)  ≤ 18.5 vs > 18.5 | | 0.509 | | 0.081-9.790 | | 0.569 | |  | |  | |  | |  | |
| Body mass index (kg/m2)  ≤ 25.0 vs > 25.0 | | - | | - | | 0.139 | |  | |  | |  | |  | |
| Body mass index (kg/m2)  ≤ 30.0 vs > 30.0 | | - | | - | | 0.507 | |  | |  | |  | |  | |
| Disease specific survival | | | | | | | | | | | | | | | |
|  | | Univarite analysis | | | | | |  | | Multivariate analysis | | | | | |
| Parameters | | Hazard ratio | | 95% CI | | *p* value | |  | | Hazard ratio | | 95% CI | | *p* value | |
| Age at opetation (yr)  ≤ 60 vs > 60 | | - | | - | | 0.075 | |  | |  | |  | |  | |
| Tumor size (mm)  ≤ 20.0 vs > 20.0 | | - | | - | | 0.568 | |  | |  | |  | |  | |
| Skin infiltration  Negative vs Positive | | - | | - | | 0.281 | |  | |  | |  | |  | |
| Lymph node status  Negative vs Positive | | - | | - | | 0.126 | |  | |  | |  | |  | |
| Ki67  ≤14 % vs >14 % | | 0.282 | | 0.013-2.943 | | 0.282 | |  | |  | |  | |  | |
| Objective response rate  Non-Responders vs Responders | | 0.179 | | 0.017-3.845 | | 0.217 | |  | |  | |  | |  | |
| Pathological response  Non-pCR vs pCR | | 0.539 | | 0.025-5.657 | | 0.605 | |  | |  | |  | |  | |
| TILs  Low vs High | | 1.089 | | 0.104-23.469 | | 0.945 | |  | |  | |  | |  | |
| Body mass index (kg/m2)  ≤ 18.5 vs > 18.5 | | 0.179 | | 0.017-3.845 | | 0.217 | |  | |  | |  | |  | |
| Body mass index (kg/m2)  ≤ 25.0 vs > 25.0 | | - | | - | | 0.290 | |  | |  | |  | |  | |
| Body mass index (kg/m2)  ≤ 30.0 vs > 30.0 | | - | | - | | 0.643 | |  | |  | |  | |  | |

HER2BC: human epidermal growth factor receptor 2-enriched breast cancer (ER-, PgR-, and HER2+). CI: confidence intervals. pCR: pathological complete response. TILs: tumor-infiltrating lymphocytes.
